# Real-world data to support post-market safety and performance of embolization coils: evidence generation from a medical device manufacturer and data institute partnership

**DOI:** 10.1186/s12911-024-02659-0

**Published:** 2024-09-19

**Authors:** Amelia Hochreiter-Hufford, Jennifer Gatz, Amy M. Griggs, Ryan D. Schoch, Kimberly M. Birmingham, Christopher Frederick, John Price, Scott Snyder

**Affiliations:** 1 Cook Research Incorporated, 1 Geddes Way, West Lafayette, IN 47906 USA; 2 3Aware, Indianapolis, IN USA; 3 Regenstrief Institute, Indianapolis, IN USA

**Keywords:** Real-world data, Real-world evidence, Post-market surveillance, Electronic health records, Embolization coils, Post-market clinical follow-up, Total product lifecycle, Medical devices, Unstructured data, Health information exchange

## Abstract

**Background:**

Recognizing the limitations of pre-market clinical data, regulatory authorities have embraced total product lifecycle management with post-market surveillance (PMS) data to assess medical device safety and performance. One method of proactive PMS involves the analysis of real-world data (RWD) through retrospective review of electronic health records (EHR). Because EHRs are patient-centered and focused on providing tools that clinicians use to determine care rather than collecting information on individual medical products, the process of transforming RWD into real-world evidence (RWE) can be laborious, particularly for medical devices with broad clinical use and extended clinical follow-up. This study describes a method to extract RWD from EHR to generate RWE on the safety and performance of embolization coils.

**Methods:**

Through a partnership between a non-profit data institute and a medical device manufacturer, information on implantable embolization coils’ use was extracted, linked, and analyzed from clinical data housed in an electronic data warehouse from the state of Indiana’s largest health system. To evaluate the performance and safety of the embolization coils, technical success and safety were defined as per the Society of Interventional Radiology guidelines. A multi-prong strategy including electronic and manual review of unstructured (clinical chart notes) and structured data (International Classification of Disease codes), was developed to identify patients with relevant devices and extract data related to the endpoints.

**Results:**

A total of 323 patients were identified as treated using Cook Medical Tornado, Nester, or MReye embolization coils between 1 January 2014 and 31 December 2018. Available clinical follow-up for these patients was 1127 ± 719 days. Indications for use, adverse events, and procedural success rates were identified via automated extraction of structured data along with review of available unstructured data. The overall technical success rate was 96.7%, and the safety events rate was 5.3% with 18 major adverse events in 17 patients. The calculated technical success and safety rates met pre-established performance goals (≥ 85% for technical success and ≤ 12% for safety), highlighting the relevance of this surveillance method.

**Conclusions:**

Generating RWE from RWD requires careful planning and execution. The process described herein provided valuable longitudinal data for PMS of real-world device safety and performance. This cost-effective approach can be translated to other medical devices and similar RWD database systems.

## Background

Medical device manufacturers must demonstrate that medical devices are safe and perform as intended to gain and maintain market approval. While clinical investigations are highly controlled to generate evidence for a specific clinical purpose, they are generally limited in the number of participants and duration of follow-up. Additionally, the strict inclusion and exclusion criteria of most trials may also result in a study cohort that is not entirely representative of the larger general population. Therefore, to ensure the continued safety and performance of devices after introduction into the market, post-market surveillance (PMS), as defined under Sect. 522 of the Federal Food, Drug, and Cosmetic Act [1], may require the assessment of a medical device’s benefit-risk ratio throughout the total product lifecycle. Similarly, Chapter VII of the European Union Medical Device Regulation (EU MDR) requires manufacturers to establish a PMS system for quality, performance, and safety monitoring throughout the entire lifetime of the device [2].

One method used to assess the safety and performance of devices is to use real-world data (RWD); that is, data related to patient health status or delivery of health care routinely collected from a variety of sources as part of standard medical practice [3]. This RWD, if relevant and reliable, can then be analyzed in the context of usage, benefits, and risks to generate real-world evidence (RWE), to support regulatory decision-making [4].

Sources of RWD include electronic health records (EHR), registries, administrative claims databases, patient-generated data, public health surveillance systems, medical device data repositories, among others [3]. These data sources allow for PMS through the systematic collection of information and outcomes derived from real-world clinical care, including the detection of changes in how the device is used in practice or the identification of safety signals that warrant investigation. RWD may also allow for assessment of long-term outcomes. Traditionally, clinical studies with multi-year follow-up requirements are burdensome to patients and require significant resources to execute. Conversely, longitudinally tracked patient data collected over their continuum of care not only reduces patient burden and loss-to-follow-up, but may also decrease the time required to obtain data on long-term safety and performance. Retrospectively-obtained patient RWD can contain years of follow-up and thereby expedite both regulatory decision-making and identification of safety signals, instead of waiting for such data in real-time [3]. Furthermore, RWD collection efforts can be cost-effective compared with more traditional methods of conducting a post-market clinical study [5].

However, the generation of RWE can be challenging, due to variability in the quality, reliability, and relevance of the RWD found in these sources [6]. Common challenges in RWD include inconsistent data coding, missing information, lack of follow-up data [7], the need for careful data quality reviews, as well as concerns regarding the protection of patient privacy [6]. Additionally, statistical analyses must be carefully planned and applied to RWD to avoid inappropriate conclusions when generating RWE.

This study describes the methods used to extract RWD on patients treated with implantable embolization coils from an EHR database. This data was then compared with rates of outcomes specified in the literature to generate RWE on the ongoing safety and performance of these medical devices.

## Methods

### Aim and design

This retrospective, observational, post-market data collection was conducted between April 2020 – June 2021 with the aim to verify the continued safety and performance of Cook implantable embolization coils; specifically, the Tornado^®^ Embolization Coils and Microcoils, MReye^®^ Embolization Coils, and Nester^®^ and MicroNester^®^ Embolization Coils (Cook Incorporated, Bloomington, IN). The intended use of these devices, at the time of the study, was for arterial and venous embolization in the peripheral vasculature in adult patients.

Cook Group Incorporated, an Indiana-based medical device company, has international regulatory requirements for reporting on the safety and performance of Cook products. To collect post-market device data, Cook partnered with the Regenstrief Institute, an Indiana-based non-profit research institute. A data extraction protocol was submitted by the Regenstrief Institute to the Indiana University (IU) Institutional Review Board (IRB) requesting exemption of informed consent in accordance with 45 CFR 46.101(b) and/or IU Human Research Protection Program Policy, and exemption was granted. All data were de-identified before analysis following the HIPAA safe harbor method.

### Study population

For each of the embolization coil types, a minimum of 100 patients who received an implantable embolization coil and had at least one additional clinical encounter 30 days or more following the implantation were required, to ensure that patients had a record of receiving care within the included data sources. The inclusion process started with calendar year 2014 and proceeded forward in time through December 2018, to allow for the majority of patients to have over 1 year of follow-up information. This resulted in inclusion time periods that varied among the three embolization coil types. Patients were excluded from further data analysis if an operative report was not present within the health information exchange or if no post-encounter data were available. As embolization coils are permanent implanted devices, long-term safety assessment required the inclusion of patients with multi-year longitudinal follow-up. Because of the retrospective nature of the data collection and the variable clinical indications for use represented in this study, the length of time from the index procedure to the final post-procedure encounter varied per patient.

### Data sources

Via the Regenstrief Institute, data from patient populations were extracted from the Enterprise Data Warehouse (EDW) of a major Indiana-based healthcare system, which contains healthcare and device-related data within multiple linked tables from system-associated inpatient and outpatient facilities within Indiana. Follow-up patient data were gathered from both the EDW and the Indiana Network for Patient Care (INPC) database. The INPC database is managed by the Indiana Health Information Exchange (IHIE). IHIE is one of the largest health information exchange networks in the United States. It is a large data repository that contains clinical data from 38 health systems, mostly within the state of Indiana. The models and catalog numbers for the devices of interest were gathered from the National Institutes of Health (NIH) website (https://accessgudid.nlm.nih.gov/) and Cook Medical’s website (https://www.cookmedical.com/products/).

### Identification of devices of interest

**Fig. 1:**
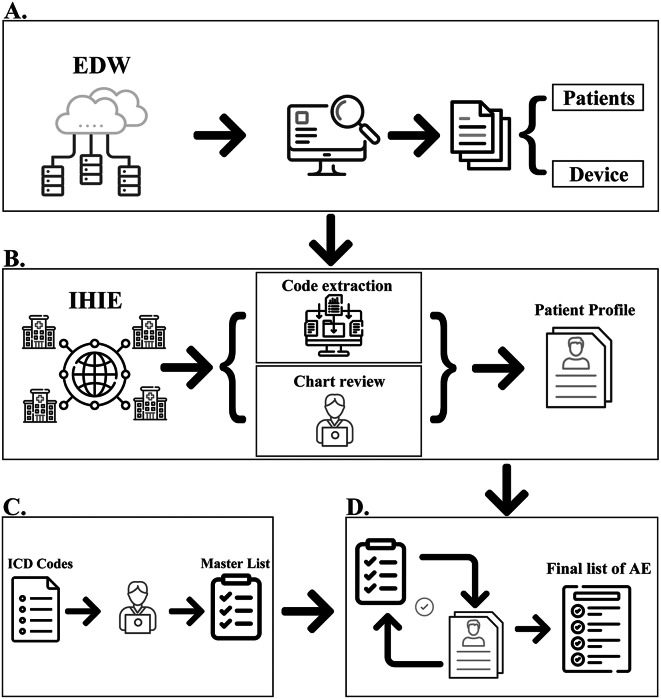
**Dataset creation.** First device use was initially identified in the Electronic Data Warehouse (EDW), where the index procedure data (including identification of the patient and the device(s) implanted) was extracted (**A**). The surgical case was then linked to the information available on the same patient in the Indiana Health Information Exchange (IHIE) system. All available diagnosis codes from both the index procedure and adverse events of interest were then extracted from the IHIE system via automated CPT code extraction and manual review of the patient charts as well as operative notes for any mention of embolization coils. These data were collated to create patient profiles for each of the patients identified (**B**). The profiles were compared with a master list of adverse event codes of interest generated from review of the United States’ Center for Medicare and Medicaid (CMS) diagnosis code list (**C**) to obtain the final list of adverse events related to the embolization procedures for each patient in this review (**D**)

For this study, searches were conducted via the “Implantation” table within the EDW to identify first device use (Fig. 1A). The EDW contains an implant log summary table which stores information on devices used during surgical procedures, such as manufacturer and model number. A record in the implant log summary table was linked to a surgical case, which was then linked to a patient and a clinical encounter (e.g. hospital stay) in the IHIE (Fig. 1B). The models and catalog numbers of interest were used to identify the first device implant surgery per patient (index event); all diagnoses and procedures from the same clinical encounter as the index event were extracted.

### Definitions

The index event was defined as the first clinical encounter with a surgical event involving implantation of at least one of the embolization coils, identified using the device model numbers for each of the three coil types. A clinical encounter was defined as a patient’s interaction with the healthcare system, such as an inpatient stay or outpatient procedure. The encounter links together the clinical data captured during the patient’s interaction.

Safety was defined as the occurrence of pre-specified major adverse events within 30 days of, and related to, the embolization procedure. Events that resulted in an unplanned increase in the level of care, prolonged hospitalization, or permanent adverse sequelae were also considered to be major adverse events. Adverse events of interest were pre-specified based on the known device risks identified in the manufacturer’s Instructions for Use (IFU), the Society of Interventional Radiology (SIR) quality improvement guidelines [8], or potential complications associated with device placement. These pre-specified major adverse events included target vessel ischemia, non-target embolization, coil migration, sepsis, abscess, hemorrhage, spinal infarction, access site complication, and procedure-related death. The acceptable rate for safety events was set at 12% or less based on quality improvement guidelines published by the SIR [8]. Safety events were identified both through structured and unstructured data.

Technical success was defined as cessation or restriction of blood flow to the target area following coil deployment, ascertained through manual review of the charts for specific mentions of bleeding cessation after the index procedure. An acceptable rate for technical success of 85% or greater was set based on quality improvement guidelines published by the SIR [8].

An assumption of technical success was made in a subset of patients for whom the chart did not specifically mention bleeding cessation or restriction but described a continuation or completion of the index procedure with no notes of additional bleeding events that suggest a technical failure and no evidence of a subsequent procedure within the follow-up period.

### Dataset creation and review

Figure 1 depicts a summary of the dataset creation process; a novel approach for obtaining real-world data on medical devices that include identification of the device, as well as obtaining information on procedural outcomes and adverse events structured and unstructured data. The index date for each patient was the recorded date of the device implantation from the EDW system as described above. Structured codes for these elements included International Classification of Disease (ICD)-9, ICD-10 and Current Procedural Terminology (CPT)-4 codes used by the hospitals. Corresponding diagnosis, procedure, patient age and sex, and encounter information for that patient were also extracted from the EDW system (Fig. 1A). Patients were then identified within the IHIE dataset, where all subsequent diagnoses, procedures, and encounter information from institutions that have consented to research were extracted via automated code extraction. In addition to these automated queries, chart reviews of the index operative report were conducted to extract anatomic location and indication for coil placement, number of coils placed, and technical success. This review of available index procedure unstructured data was used to supplement the clinical data extracted from the EDW and identify indication for use (Fig. 1B).

To evaluate short-term safety events, available diagnosis codes listed at the time of the index procedure were first extracted for each patient to assess potential comorbidities. These extracted diagnosis codes were then organized into their respective ICD-9 or ICD-10 chapter or organization to determine the indication for coil placement. Pre-specified adverse events of interest were identified using searches in the IHIE database. ICD codes were used to search the structured data, and text wildcards of interest were used to query clinical notes within the IHIE database. Furthermore, unstructured data from the medical notes post-index procedure were reviewed for the pre-selected safety events, and any mention of the embolization coils in the patient charts were also reviewed to identify any other events that were attributed to the use of the embolization coils. Extracted data were included in the final analytical dataset (Fig. 1B).

To evaluate long-term safety, post-encounter diagnosis codes of events that occurred beyond 30 days after the index procedure were reviewed were anonymized and compiled into patient profiles for each of the patients included in this study (Fig. 1B).

The information contained in each of the patient profiles was then cross-referenced to the master list of adverse events to identify events that could be related to the use of embolization coils (Fig. 1D). Each of these events was then manually reviewed by the medical professional to determine if the coils were the cause of the event or if an alternative explanation was likely. If no alternative was found, or if the event was attributed to the use of coils in the patient’s record, the adverse event was categorized as related to the use of embolization coils.

### Data analysis

Statistical analyses were performed using SAS^®^ (version 9.4; SAS Institute, Cary, NC, USA) for Windows^®^. Unless noted otherwise, continuous variables were reported as mean ± standard deviation. Categorical variables were reported as percentages and frequencies.

## Results

### Patient cohort

**Fig. 2:**
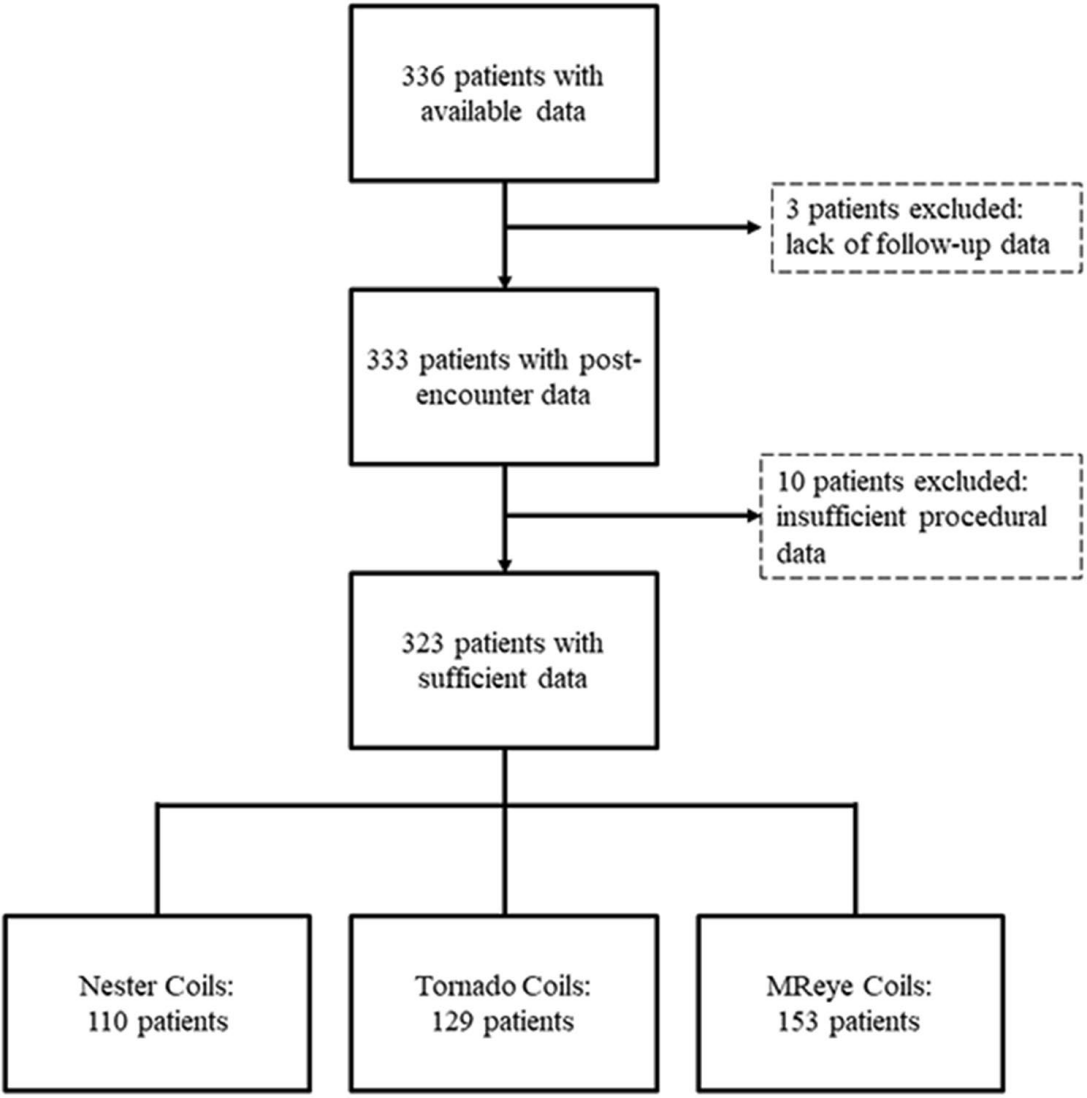
**Patient screening and inclusions.** Patients could be treated with one or more types of coils. Patients treated with more than one type of coil are represented in more than one group

A total of 336 patients who were treated using one or more Tornado, MReye, or Nester Embolization Coils from 1 January 2014 to 31 December 2018 were identified in the EDW system for initial data extraction. Upon data review, 3 patients lacked follow-up data after the index procedure and 10 patients had insufficient data regarding indication for use and location of coil deployment. Thus, 323 patients were included in the final dataset (Fig. 2). Of these, 110 patients were treated using Nester Embolization Coils, 129 were treated using Tornado Embolization Coils, and 153 were treated with MReye Embolization Coils, with 67 patients receiving more than 1 type of embolization coil and being included in more than 1 cohort (Table 1).

**Table 1:** Distribution of patients per coil type

Devices	Total patients
Nester Only	50
Tornado Only	66
MReye Only	140
Nester and Tornado	54
Nester and MReye	4
Tornado and MReye	7
Nester, Tornado, and MReye	2

The duration of follow-up varied per patient, based on the final encounter data available. The mean follow-up for the identified patients was 1127 ± 719 days; with the longest follow-up duration being 6.8 years (range: 3-2500 days).

### Indications for use

Identification of the reason for coil placement (indication for use) was completed for each of the coil types through automated mining of structured data codes as well as review of index procedure unstructured data. The frequency of each indication for use per coil type is presented in Table 2.

The most commonlyfound indications for use were malignancy (30.9%, 34/110) for the Nester Embolization Coils, malfunctioning fistula for renal dialysis (24.0%, 31/129) for the Tornado Embolization Coils, and vessel placement due to congenital cardiac defect (47.7%, 73/153) for the MReye Embolization Coils.

**Table 2:** Indication for use by coil type

Indication for use	Patients by Coil Type (%, *n*/*N*)			
	Nester	Tornado	MReye	TOTAL^a^
Aneurysm (Thoracic, Abdominal, Others)	14.5 (16/110)	11.6 (15/129)	2.6 (4/153)	7.4 (24/323)
Biliary (Non-Vessel)	0 (0/110)	8.5 (11/129)	0 (0/153)	3.4 (11/323)
Defunctionalized Bladder Flap; Recurrent UTI (Non-Vessel)	0.9 (1/110)	0.8 (1/129)	0 (0/153)	0.3 (1/323)
GI^b^ Hemorrhage	4.5 (5/110)	3.9 (5/129)	0 (0/153)	2.2 (7/323)
Hemorrhage	3.6 (4/110)	3.1 (4/129)	0 (0/153)	1.9 (6/323)
Hemorrhage Due to Injury	1.8 (2/110)	3.9 (5/129)	0 (0/153)	1.5 (5/323)
Malfunctioning Fistula for Renal Dialysis	12.7 (14/110)	24.0 (31/129)	16.3 (25/153)	18.3 (59/323)
Malignancy	30.9 (34/110)	22.5 (29/129)	0 (0/153)	13.9 (45/323)
Patent Ductus Arteriosus	0 (0/110)	0 (0/129)	32.7 (50/153)	15.5 (50/323)
Peripheral Vascular Disorder	3.6 (4/110)	3.1 (4/129)	0.7 (1/153)	1.9 (6/323)
Pseudoaneurysm	12.7 (14/110)	10.1 (13/129)	0 (0/153)	5.3 (17/323)
Scrotal Varices	0.9 (1/110)	0.8 (1/129)	0 (0/153)	0.3 (1/323)
Upper GI Ulcers	3.6 (4/110)	3.9 (5/129)	0 (0/153)	2.2 (7/323)
Varices (Portal Hypertension)	9.1 (10/110)	3.9 (5/129)	0 (0/153)	3.4 (11/323)
Vessel (E.G. Collateral) Placement Due to Congenital Cardiac Defect	0.9 (1/110)	0 (0/129)	47.7 (73/153)	22.6 (73/323)

^a^ Patients with more than one type of coils placed are represented in each of the coil type’s cohorts but counted only once for the total patient study population

^b^ Gastrointestinal

### Creation of safety events master list

ICD-9 or ICD-10 major categories available in the United States’ Center for Medicare and Medicaid Services CMS’ list were initially reviewed to determine their possible relatedness to the procedure or the embolization coils [9]. The codes were first evaluated at the level of their CMS Chapter designation. Based on known risks for embolization as per the devices’ IFU and the SIR guidelines [8], Chapters II-VII and Chapters XII-XVII were deemed to be unrelated to the embolization procedures and were therefore excluded. For the remaining Chapters, the subclassifications (Code Ranges) were then evaluated for potential relatedness to the procedure or embolization coils.

**Fig. 3:**
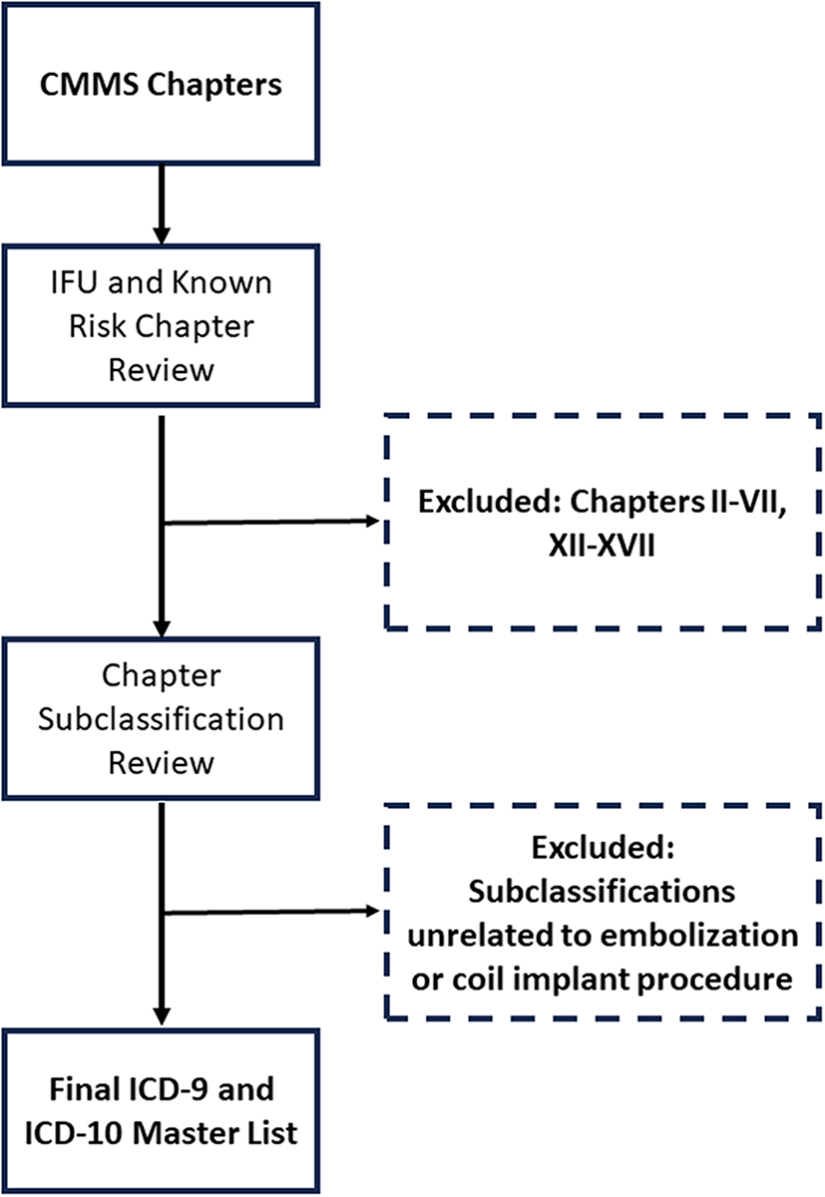
Generation of ICD Code Master List. CMS Chapters were initially reviewed based on known device usage and risks. Chapters that were unrelated to embolization procedures were excluded. Next, Chapter Subclassifications that were unrelated to embolization or coil implant procedures were also excluded. The remaining codes were made into a final Safety Events master list to cross-reference patient files

Subclassifications found to be unrelated to the embolization procedure or the use of embolization coils (e.g. mental disorders, complications of pregnancy, childbirth, and the puerperium, and congenital anomalies) were then excluded from further evaluation. From the remaining categories, a final master list of 365 ICD-9 and ICD-10 codes considered to be possibly related to embolization procedure or the use of embolization coils (Fig. 3) was finalized and used for cross-reference to all patient profiles.

### Safety and performance measures

The master list of ICD codes was used to identify safety events related to the use of embolization coils in the patient profiles. Over 40,000 ICD codes obtained from patient unstructured data were cross-referenced against this master list to detect safety events that were likely related to the device or the coil placement procedure.

Early (within 30 days of index procedure) safety events related to the use of embolization coils were identified in 5.3% of patients (17/323; 95% CI: 3.1%, 8.3%) with a total of 18 major adverse events detected in 17 patients. A list of the major adverse events identified is presented in Table 3. No deaths as a direct result of the index procedure were recorded in this dataset.

For the evaluation of longer-term safety outcomes, patient follow-up data were assessed as available, by cross-reference of the patient profiles to the ICD master list. Five long-term adverse events related to embolization coil placement, or the index procedure were identified in 5 patients (1.5%, 5/323). These included one instance of unintended embolization, one case of sepsis, one case of thrombosis, one instance of injury to the iliac artery and iliac artery transection, and one instance of an unspecified complication of vascular prosthetic device that could not be ruled out as unrelated to the embolization coils (Table 3). Thus, the safety goal of an adverse event rate at or below 12% set for this study based on SIR guidelines was successfully met.

**Table 3:** Adverse events related to embolization coil use

Adverse event	Adverse Events for Patients by Coil Type (%, *n*/*N*)			
	Nester	Tornado	MReye	TOTAL
**MAJOR ADVERSE EVENTS (WITHIN 30 DAYS)**				
Abscess	0 (0/110)	0.8 (1/129)	0 (0/153)	0.3 (1/323)
Cardiac arrest	0.9 (1/110)	0 (0/129)	0 (0/153)	0.3 (1/323)
Embolization	4.5 (5/110)	4.7 (6/129)	1.3 (2/153)	2.5 (8/323)
Hemorrhage	0 (0/110)	0 (0/129)	0 (0/153)	0 (0/323)
Infarction	0 (0/110)	0 (0/129)	0 (0/153)	0 (0/323)
Ischemia	0 (0/110)	0 (0/129)	0 (0/153)	0 (0/323)
Migration	1.8 (2/110)	1.6 (2/129)	0.7 (1/153)	0.9 (3/323)
Procedure-related death	0 (0/110)	0 (0/129)	0 (0/153)	0 (0/323)
Sepsis	0 (0/110)	0 (0/129)	0.7 (1/153)	0.3 (1/323)
Stroke	0 (0/110)	0.8 (1/129)	0 (0/153)	0.3 (1/323)
Others^a^	2.7 (3/110)	0.8 (1/129)	0 (0/153)	0.9 (3/323)
**Overall Major Adverse Events**	**9.1 (10/110)**	**7.8 (10/129)**	**2.6 (4/153)**	**5.3 (17/323)**
**LONG-TERM ADVERSE EVENTS**				
Abscess	0 (0/110)	0 (0/129)	0 (0/153)	0 (0/323)
Embolization	0 (0/110)	0.8 (1/129)	0 (0/153)	0.3 (1/323)
Hemorrhage	0 (0/110)	0 (0/129)	0 (0/153)	0 (0/323)
Infarction	0 (0/110)	0 (0/129)	0 (0/153)	0 (0/323)
Ischemia	0 (0/110)	0 (0/129)	0 (0/153)	0 (0/323)
Migration	0 (0/110)	0 (0/129)	0 (0/153)	0 (0/323)
Procedure-related death	0 (0/110)	0 (0/129)	0 (0/153)	0 (0/323)
Sepsis	0 (0/110)	0.8 (1/129)	0 (0/153)	0.3 (1/323)
Thrombosis	0 (0/110)	0 (0/129)	0.7 (1/153)	0.3 (1/323)
Others^b^	1.8 (2/110)	0.8 (1/129)	0 (0/153)	0.6 (2/323)
**Overall LongTerm Adverse Events**	**1.8 (2/110)**	**2.3 (3/129)**	**0.7 (1/153)**	**1.5 (5/323)**

^**a**^Includes one instance of limb swelling, limb pain, and abdominal pain after infrarenal abdominal aortic aneurysm repair that could not be disregarded in relatedness to the embolization coils and two instances of acute kidney failure

^b^Includes one instance of injury to the iliac artery and iliac artery transection and one instance of an unspecified complication of a vascular prosthetic device, implants, and grafts that could not be disregarded in relatedness to the embolization coils

Performance of the medical devices was identified via chart review to determine technical success, as defined previously. A total of 20 patients were excluded from technical success estimates; 8 patients were excluded due to lack of coil location data and 12 patients were excluded due to non-vascular placement. Out of the remaining 303 patients, information on technical success as defined for this study was identified for 258 patients. For 45 patients, an indication of technical success was not readily identified in the physician notes after manual review. Thus, for these 45 patients an assumption of technical success was made based on the continuation or completion of the index procedure without the mention of additional bleeding events. Technical success by indication for use and coil type is presented in Table 4.

A total of 10 technical failures were detected as part of the review. These failures occurred in 2 patients treated with Nester Embolization Coils, 3 patients treated with Tornado Embolization Coils, 1 patient treated with MReye Embolization Coils, 3 patients treated with both Nester and Tornado Embolization Coils, and 1 patient treated with both Tornado and MReye Embolization Coils. Reasons for failure included coil malposition, coil migration that required intervention, coil size being too small for the target anatomy, failure to occlude the intended vessel/failure to stop bleeding, coil prolapse into hepatic artery that required removal, and bleeding duodenal ulcer post esophagogastroduodenoscopy and embolization requiring laparotomy.

Technical success was 96.7% for the overall cohort (293/303; 95% CI: 94.0%, 98.4%), with 95.4% (104/109), 94.0% (110/117), and 98.6% (143/145) for the Nester, Tornado, and MReye cohorts, respectively. Thus, the performance goal of technical success 85% or greater set for this study based on SIR guidelines was successfully met through this real-world dataset.

**Table 4:** Technical success by indication for use and coil type

Indication for use	Technical Success for Patients by Coil Type (%, *n*/*N*)			
	Nester	Tornado	MReye	TOTAL^a^
Aneurysm (Thoracic, Abdominal, Others)	100.0 (16/16)	100.0 (15/15)	100.0 (4/4)	100.0 (24/24)
GI^b^ Hemorrhage	100.0 (5/5)	100.0 (5/5)	N/A	100.0 (7/7)
Hemorrhage	100.0 (4/4)	100.0 (4/4)	N/A	100.0 (6/6)
Hemorrhage Due to Injury	100.0 (2/2)	100.0 (5/5)	N/A	100.0 (5/5)
Malfunctioning Fistula for Renal Dialysis	100.0 (14/14)	96.8 (30/31)	96.0 (24/25)	98.3 (58/59)
Malignancy	91.2 (31/34)	93.1 (27/29)	N/A	93.3 (42/45)
Patent Ductus Arteriosus	N/A	N/A	98.0 (49/50)	98.0 (49/50)
Peripheral Vascular Disorder	100.0 (4/4)	75.0 (3/4)	100.0 (1/1)	83.3 (5/6)
Pseudoaneurysm	92.9 (13/14)	92.3 (12/13)	N/A	94.1 (16/17)
Scrotal Varices	100.0 (1/1)	100.0 (1/1)	N/A	100.0 (1/1)
Upper GI Ulcers	100.0 (4/4)	60.0 (3/5)	N/A	71.4 (5/7)
Varices (Portal Hypertension)	90.0 (9/10)	100.0 (5/5)	N/A	90.9 (10/11)
Vessel (E.G. Collateral) Placement Due to Congenital Cardiac Defect	100.0 (1/1)	N/A	100.0 (65/65)	100.0 (65/65)

^a^A total of 67 patients being treated with more than one coil type; N/A, not applicable as no coils of that type were placed for that particular indication

^b^ Gastrointestinal

## Discussion

Medical device manufacturers must implement innovative ways of conducting PMS to ensure safety and performance over the lifetime of their products. Here, we describe a method for extracting RWD from EHRs to support safety and performance assessments for permanent implantable embolization coils.

EHRs can be a powerful source of RWD for medical device use, informing on patient health status outside of the strict parameters set forth by traditional clinical trials [3, 10, 11]. However, before embarking on a RWD study, sponsors must carefully design the study to ensure that the RWE generated will be scientifically valid and adequately support their regulatory needs. The recent US FDA guidance: “Use of Real-World Evidence to Support Regulatory Decision-Making for Medical Devices”, issued on December 19, 2023, provides the most comprehensive outline of expectations to date. The European Union’s Medical Device Coordination Group (MDCG) has yet to explicitly provide similar guidance for regulatory decision-making in Europe, but the expectation for determining quality and reliability is stated in MDCG 2020-7 [12]. 

While device manufacturers can undertake assessments of relevance such as data availability, data linkage, timeliness, and generalizability of RWD, collaborations between device manufacturers and data institutes with access to EHR data, such as the one presented in this study, is required to assess reliability of the data. Data institutes should disclose key elements such as data transformations (including those made for privacy protection), data completeness, data cleaning and cross-referencing procedures, and auditing rules, methods, and mitigation strategies to reduce error. Without this level of disclosure and collaboration, device manufacturers are unable to ensure the quality and scientific validity expected by regulatory authorities [3, 5]. 

If relevance and reliability can be confirmed, RWD may provide powerful insights not available with other sources. RWD can provide insights into device safety and performance across more heterogeneous patient populations than traditional study methods. While this heterogeneity can be seen as a limitation of RWD [5], it can provide a more comprehensive understanding of the risks and performance of a product in clinical practice if selection bias is monitored.

Detection of off-label use is not possible with more traditional methods of clinical research. Even some sources of RWD, such as registries, are not likely to capture off-label use if treatment is prospectively defined under a clinical protocol. However, while not yet specifically mandated by the US FDA, the EU MDR Annex XIV part B now requires manufacturers, as an aim of post-market clinical follow-up, to proactively determine if systemic off-label use or misuse occurs [2]. Through the method presented in this manuscript, we identified instances of non-vascular placement of embolization coils and identified pediatric use of the MReye embolization coils for the treatment of congenital heart defects, both of which are outside of the devices’ intended use of embolization in the peripheral vasculature. While the intended use of these devices at the time of the study did not address pediatric use, the intended patient population has since been updated to specify an adult patient population. The instances of use of the MReye Embolization Coils in a pediatric population within this study all came from a single children’s hospital affiliated with the EDW system, which explains the observed use of these coils in pediatric patients. Traditional methods of clinical research in which study protocols specify treatment methods may not have captured this use of coils within pediatric patients.

The potential for using EHRs for the collection of RWD on medical devices has been reported by several other groups as part of the National Evaluation System for Health Technology (NEST), an initiative in which the Food and Drug Administration (FDA) seeks to provide detailed information regarding medical devices in real-world settings [13]. Druva et al. [14] used EHR data to support premarket safety information and possible label expansion for a thermal ablation catheter. For that study, manual review added a degree of quality assurance for EHR data extracted by a Large Language Model developed by the authors [15]. Barnes et al. [16] reported on the creation of an aggregate database from several registries using hashtag variables, allowing them to conduct a feasibility study of off-label combinations of endoprostheses for the treatment of iliac artery aneurysms based entirely on the review of RWD. These studies highlight approaches that can ease the review of these large databases through automation of the data collection and outline the potential of RWE generation to inform regulatory decisions.

The access to and analysis of device-specific RWD can also be cost- and time- effective. In the present study, years of patient-specific data from multiple clinical sites were extracted, reviewed, and analyzed in just over a year, for a fraction of the cost of a traditional clinical study. Similar efforts to conduct a post-market, multi-site clinical trial to generate evidence on the continued safety and performance of a device could be cost-prohibitive for some devices or companies. A 2018 investigation estimated the average cost of a post-approval study for a complex medical device to be around $6 million and span an average of 81 months [17]. A different study estimated that over an 8-year period ranging from March 2005-June 2013, the median cost for an FDA-mandated post-approval study was more than $2.1 million [18]. In contrast, a 2020 study comparing the potential value of leveraging RWD from the Society for Vascular Surgery Vascular Quality Initiative (VQI) registry to independent industry studies found that the use of the RWD in the registry resulted in cost savings of 59% on per-patient cost as well as enrollment time savings of 45-71% [19].

Still, the collection of RWD and the transformation into RWE can be challenging. RWD may not have the same quality controls as data collected within a clinical trial. Additionally, the transformation of RWD into RWE continues to be a challenge due to the lack of a Universal Device Identifier (UDI) included within patient health care records. Additional potential challenges include missing data [7, 20], selection bias [7, 20], lack of follow-up information [7], absence of controls, and multiple hypothesis testing and increased type-I error rate [20]. The lack of consistent terminology or data coding can create issues with perceived reproducibility, rigor, and confidence in the data when extracted from healthcare databases [21]. For these reasons, device manufacturers considering a real-world study design should carefully assess their study questions against the available dataset and partner with data providers able to support their justification of sufficient quality.

Another limitation in the use of EHRs involves the difficulty of longitudinal follow-up of in these databases [7]. As the United States lacks a unified national healthcare database, EHRs are often limited to data from a single healthcare system or registry. Thus, if a patient obtains care outside of those centers, that data will not be captured as part of the RWD extraction efforts. In our study, we tried to minimize the effect of this limitation by using data from a large HIE (which includes multiple health systems) to collect post-procedure clinical encounters. Nevertheless, it is possible that the patients included in this dataset also received healthcare outside of these networks that was not captured in the data reviewed. Incorporating data sources that have access to multiple health systems that can longitudinally track patients across care sites could help reduce this limitation.

The under-reporting of commercial-use device failures and adverse events to regulatory authorities is well known. Therefore, proactively collecting safety and performance data through post-market clinical follow-up activities may allow for better characterization of potential risks [22–24]. The method presented in this study presents challenges, including the difficulty of locating the medical devices used in the procedures from an EHR as well as the need for extensive manual review, with over 600 hours of manual labor needed to compile and analyze the data reported above. Still, in assessing the structured and unstructured data available from patient EHRs this method was able to capture performance failures and adverse events, with rates that align with what is expected from treatment guidelines [8]. 

Finally, device manufacturers must consider how to query larger datasets, especially with multi-year, patient-level datasets, for detecting adverse events. While setting limits on the scope of the data, by using pre-specified adverse events of interest, allows for focused data mining of the large datasets common in RWD, there is the potential of a patient presenting an adverse event unknown to the manufacturer that is missed through this strategy. In our study, we lessened this effect by also conducting a manual review of the patient charts for mentions of embolization coils after the index procedure. This would identify events that had been attributed to the use of embolization coils by the practicing physician. However, events that were not directly attributed to the use of coils in the notes still would have been missed by this strategy. A more thorough review of the patient-level unstructured data would be required to increase the likelihood of unanticipated adverse events being discovered, and upcoming technologies using the assistance of artificial intelligence (AI) may increase the amount of unstructured data that can be reviewed accurately.

## Conclusions

This analysis demonstrates a method for RWE generation through a collaboration between a medical device manufacturer and a healthcare research organization. By engaging in a collaborative framework as described herein, this strategy can provide more comprehensive and resource effective information on the safety and performance of implantable devices within real-world settings. However, such analysis of RWD datasets to evaluate medical device safety and performance requires a level of manual effort, which for larger patient cohorts could benefit in using the assistance of AI. These methods contribute to the continued evolution of leveraging data available in database systems to inform clinical care.

## Data Availability

The datasets used and analyzed during the current study are available from the corresponding author upon reasonable request.

## Abbreviations

CPT: Current Procedural Terminology
CMS: United States’ Center for Medicare and Medicaid Services
EDW: Enterprise Data Warehouse
EHR: Electronic Health Records
EU MDR: European Union Medical Device Regulation
FDA: Food and Drug Administration
ICD: International Classification of Disease
IHIE: Indiana Health Information Exchange
INPC: Indiana Network for Patient Care
IRB: Institutional Review Board
IU: Indiana University
NEST: National Evaluation System for Health Technology
NIH: National Institutes of Health
PMS: Post-market surveillance
RWD: Real-world data
RWE: Real-world evidence
SIR: Society of Interventional Radiology
UDI: Universal Device Identifier
VQI: Vascular Quality Initiative
